# Múltiplas Complicações Tromboembólicas em uma Paciente com Policitemia Vera

**DOI:** 10.36660/abc.20250153

**Published:** 2025-08-06

**Authors:** Barbara Zdzierak, Bernadeta Chyrchel, Andrzej Surdacki, Stanisław Bartuś, Artur Dziewierz

**Affiliations:** 1 University Hospital Clinical Department of Cardiology and Cardiovascular Interventions Kraków Polônia Clinical Department of Cardiology and Cardiovascular Interventions, University Hospital, Kraków - Polônia; 2 Jagiellonian University Medical College Institute of Cardiology Second Department of Cardiology Kraków Polônia Second Department of Cardiology, Institute of Cardiology, Jagiellonian University Medical College, Kraków - Polônia

**Keywords:** Trombose, Infarto do Miocárdio, Policitemia Vera

## Introdução

A policitemia vera (PV) é uma neoplasia mieloproliferativa rara, negativa para o cromossomo Filadélfia, caracterizada pela proliferação clonal de células-tronco multipotentes da medula óssea, levando à eritrocitose e ao aumento da massa de glóbulos vermelhos.^1^ Pode coexistir com outros distúrbios hematológicos.^2^ A PV resulta em hiperviscosidade sanguínea, o que predispõe os pacientes à trombose — a complicação mais comum e clinicamente significativa da doença.^1^ A mielofibrose pós-PV (MF pós-PV) representa um estágio avançado na progressão natural da PV.^3^ Em pacientes com MF pós-PV, a trombose também continua sendo uma das principais complicações.^3^ Apresentamos aqui um caso de múltiplos eventos trombóticos, incluindo síndrome coronariana aguda, trombose arterial digital e acidente vascular encefálico isquêmico, em uma paciente com PV.

## Relato de caso

Mulher de 74 anos com diagnóstico de PV desde 2018, baseado em níveis elevados de hemoglobina e na presença da mutação *JAK2* , foi internada com dor torácica intensa. Seu histórico médico incluía suspeita de MF pós-PV (aguardando confirmação por biópsia de medula óssea), além de hipertensão arterial, hepatite C tratada e hepatite autoimune.

**Figura 1:**
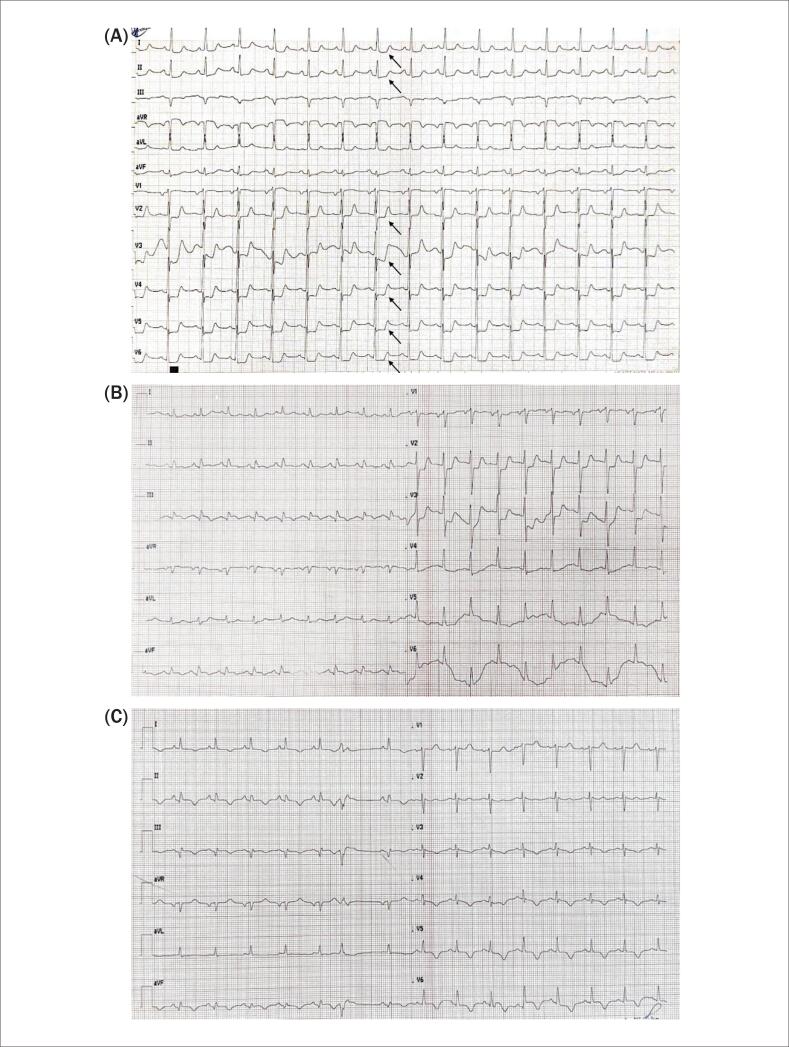
Eletrocardiograma (ECG) inicial na admissão, mostrando depressão do segmento ST nas derivações I, II e V2–V6 (setas) (A). ECG durante o episódio de edema pulmonar (B). ECG na alta hospitalar (C).

**Figura 2:**
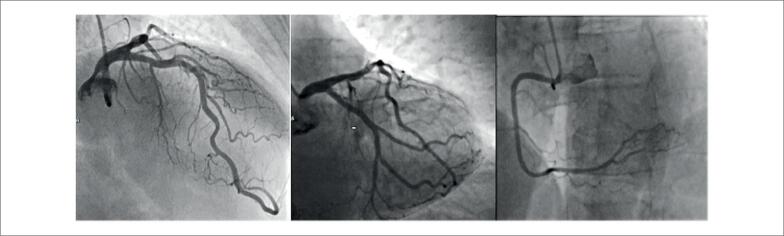
Angiografia coronariana sem alterações significativas nas artérias coronárias.

O eletrocardiograma (ECG) na admissão mostrou depressão do segmento ST nas derivações I, II e V2–V6 ( Figura 1A ). Exames laboratoriais revelaram troponina T de alta sensibilidade em 311 ng/L (valor de referência < 14 ng/L). Diante da suspeita de síndrome coronariana aguda, a paciente foi submetida a angiografia coronariana de emergência, que revelou artérias coronárias normais ( Figura 2 ). Assim, foi estabelecido o diagnóstico de infarto do miocárdio com artérias coronárias não obstrutivas (MINOCA).

**Figura 3:**
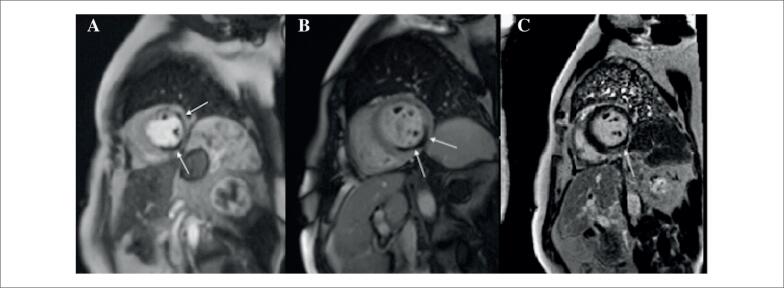
Ressonância magnética cardíaca. As setas indicam obstrução microvascular (áreas de hiporrealce) visível na perfusão de primeira passagem (A), no realce precoce pelo gadolínio (B) e no realce tardio pelo gadolínio (C).

Os exames laboratoriais também mostraram anemia moderada e trombocitose. A ecocardiografia transtorácica revelou fração de ejeção do ventrículo esquerdo discretamente reduzida (40%-42%), com hipocinesia das paredes lateral, inferolateral e inferior. A ressonância magnética cardíaca (RMC) confirmou lesões isquêmicas novas nessas regiões e evidenciou obstrução microvascular ( Figura 3 ).

Durante a internação, a paciente desenvolveu edema agudo de pulmão, necessitando de tratamento intensivo. Devido à elevação adicional dos níveis de troponina T (6562 ng/l), piora das alterações no ECG ( Figura 1B ) e queda na fração de ejeção do ventrículo esquerdo, foi realizada uma segunda angiografia coronariana, que novamente não mostrou doença obstrutiva.

**Figura 4:**
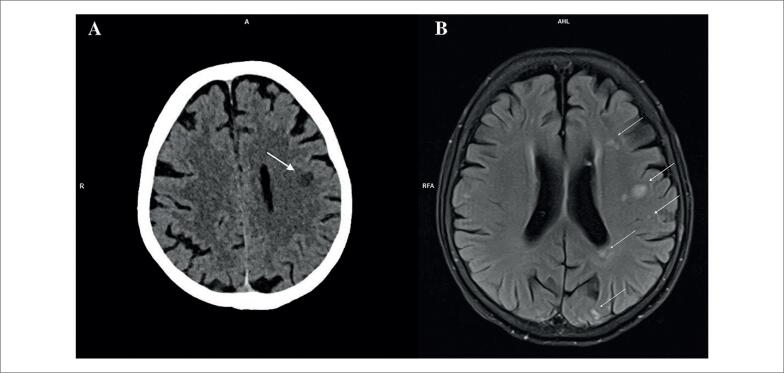
Tomografia computadorizada de crânio mostrando acidente vascular encefálico isquêmico no lobo frontal esquerdo (seta) (A). Ressonância magnética do encéfalo evidenciando múltiplas lesões isquêmicas em diferentes territórios vasculares (setas) (B).

Após estabilização clínica, a paciente relatou dor e cianose visível no segundo dedo da mão direita, sugestivos de embolia arterial digital. Iniciou-se heparina de baixo peso molecular. Pouco depois, ela desenvolveu tonturas, alterações visuais e cefaleia. A tomografia computadorizada de crânio confirmou um acidente vascular encefálico isquêmico no lobo frontal esquerdo ( Figura 4A ), manejado de forma conservadora. A ressonância magnética do encéfalo revelou múltiplas lesões isquêmicas agudas e subagudas em diferentes territórios vasculares ( Figura 4B ).

A hemoglobina da paciente caiu para 7,7 g/dL, sendo necessária transfusão sanguínea. Diante da ocorrência de múltiplos eventos trombóticos e da anemia persistente, o esquema anticoagulante foi ajustado para associação de clopidogrel com apixabana em dose reduzida (2,5 mg duas vezes ao dia). Após melhora do estado clínico, incluindo queda nos níveis de troponina T (3944 ng/l) ( Material Supplementar, Figura 1 ) e estabilização geral, a paciente recebeu alta após 16 dias de internação.

## Discussão

As neoplasias mieloproliferativas estão associadas a maior risco de eventos trombóticos, que variam de distúrbios microcirculatórios leves até tromboses arteriais e venosas graves. Além disso, a progressão da anemia agrava a lesão miocárdica devido ao desequilíbrio entre oferta e demanda de oxigênio, podendo levar ao infarto do miocárdio mesmo na ausência de obstrução arterial coronariana.^4^

O MINOCA é mais frequentemente observado em pacientes com câncer do que naqueles sem a doença.^5^ A presença do câncer piora o prognóstico tanto no MINOCA quanto no infarto com artérias coronárias obstruídas.^5^ Quando a angiografia coronariana funcional não identifica a causa subjacente do infarto, os exames de imagem não invasivos — especialmente a RMC — desempenham papel fundamental no diagnóstico.

Disponibilidade de Dados

Os conteúdos subjacentes ao texto da pesquisa estão contidos no manuscrito.

## *Material suplementar

Para informação adicional, por favor, clique aqui .
