# Supplementary material for: Múltiplas Complicações Tromboembólicas em uma Paciente com Policitemia Vera
Source: Arq Bras Cardiol. 2025 Aug 6;122(7):e20250153. [Article in Portuguese] doi: 10.36660/abc.20250153 (PMC12695099; doi:10.36660/abc.20250153)
Supplement: Translation

# Translation: Multiple Thrombotic Complications in a Patient With Polycythemia Vera

**DOI:** 10.36660/abc.20250153i

**Authors:** Barbara Zdzierak, Bernadeta Chyrchel, Andrzej Surdacki, Stanisław Bartuś, Artur Dziewierz

**Affiliations:** 1 Kraków Poland Clinical Department of Cardiology and Cardiovascular Interventions, University Hospital, Kraków – Poland; 2 Kraków Poland Second Department of Cardiology, Institute of Cardiology, Jagiellonian University Medical College, Kraków – Poland

**Keywords:** Thrombosis, Myocardial Infarction, Polycythemia Vera

## Introduction

Polycythemia vera (PV) is a rare Philadelphia chromosome–negative myeloproliferative neoplasm characterized by the clonal proliferation of multipotent bone marrow progenitor cells, leading to erythrocytosis and increased red blood cell mass.^1^It may coexist with other hematologic disorders.^2^PV results in blood hyperviscosity, which predisposes patients to thrombosis — the most common and clinically significant complication of PV.^1^Post-PV myelofibrosis (post-PV MF) represents an advanced stage in the natural progression of PV.^3^In patients with post-PV MF, thrombosis also remains one of the major complications.^3^We present a case of multiple thrombotic events, namely acute coronary syndrome, digital arterial thrombosis, and ischemic stroke, in a patient with PV.

## Case report

A 74-year-old woman with PV, diagnosed in 2018 based on elevated hemoglobin levels and the presence of a *JAK2* mutation, was admitted to the hospital with severe chest pain. Her medical history included suspected post-PV MF pending bone marrow confirmation, along with arterial hypertension, treated hepatitis C, and autoimmune hepatitis.

The admission electrocardiogram (ECG) showed ST-segment depression in leads I, II, and V2–V6 ( Figure 1A ). Laboratory testing revealed a high-sensitivity troponin T level of 311 ng/L (reference < 14 ng/L). Because of the suspicion of acute coronary syndrome, the patient underwent emergency coronary angiography, which revealed normal coronary arteries ( Figure 2 ). This led to a working diagnosis of myocardial infarction with nonobstructive coronary arteries (MINOCA).

**Figure 1:** Initial electrocardiogram (ECG) on admission showing ST-segment depression in leads I, II, and V2–V6 (arrows) (A). ECG during the episode of pulmonary edema (B). ECG at discharge (C).

**Figure 2:** Coronary angiography showing no significant abnormalities in the coronary arteries.

Laboratory results also showed moderate anemia and thrombocytosis. Transthoracic echocardiography revealed mildly reduced left ventricular ejection fraction (40%-42%) with hypokinesis of the lateral, inferolateral, and inferior walls. Cardiac magnetic resonance (CMR) imaging confirmed new ischemic injury in these regions and demonstrated microvascular obstruction ( Figure 3 ).

**Figure 3:** Cardiac magnetic resonance imaging. Arrows indicate microvascular obstruction (areas of hypoenhancement) visible on first-pass perfusion (A), early gadolinium enhancement (B), and late gadolinium enhancement (C).

During hospitalization, the patient developed acute pulmonary edema requiring intensive treatment. Due to further elevation in troponin T levels (6562 ng/L), worsening ECG changes ( Figure 1B ), and declining left ventricular ejection fraction, a second coronary angiography was performed, which again showed no obstructive coronary disease.

Following clinical stabilization, the patient reported pain and visible cyanosis in the second finger of her right hand, suggestive of digital arterial embolism. Low-molecular-weight heparin was initiated. Shortly afterward, she developed dizziness, visual disturbances, and headaches. Brain computed tomography confirmed an ischemic stroke in the left frontal lobe ( Figure 4A ), which was managed conservatively. Brain magnetic resonance imaging subsequently revealed multiple acute and subacute ischemic lesions across different vascular territories ( Figure 4B ).

**Figure 4:** Head computed tomography showing an ischemic stroke in the left frontal lobe (arrow) (A). Brain magnetic resonance imaging showing multiple ischemic lesions in different vascular territories (arrows) (B).

The patient's hemoglobin level dropped to 7.7 g/dL, requiring a blood transfusion. Due to multiple thrombotic events and persistent anemia, the patient was placed on a combination of clopidogrel and reduced-dose apixaban (2.5 mg twice daily) for anticoagulation. After improvement in her clinical status, including a decrease in troponin T levels (3944 ng/L) ( Supplementary material, Figure 1 ) and overall stabilization, the patient was discharged after a 16-day hospital stay.

## Discussion

Myeloproliferative neoplasms are associated with an increased risk of thrombotic events, ranging from mild microcirculatory disturbances to major arterial and venous thromboses. Additionally, the progression of anemia exacerbates myocardial injury due to an imbalance between oxygen supply and demand, potentially leading to myocardial infarction in the absence of obstructive coronary artery disease.^4^

MINOCA is more frequently observed in patients with cancer compared with those without cancer.^5^The presence of cancer worsens the prognosis in both MINOCA and myocardial infarction with obstructive coronary artery disease.^5^When functional coronary angiography fails to identify the underlying cause of myocardial infarction, noninvasive imaging — particularly CMR imaging — plays a crucial role in establishing the diagnosis.

Data Availability

The underlying content of the research text is contained within the manuscript.

## *Supplemental Materials

For additional information, please click here .
